# Minimally invasive mitral valve surgery: standard vs. endoscopic approach

**DOI:** 10.3389/fcvm.2026.1736030

**Published:** 2026-02-19

**Authors:** Georgios Theodosiadis, Afsaneh Karimian-Tabrizi, Tomas Holubec, Florian Hecker, Heike Strohschnitter, Thomas Walther, Fabian Emrich

**Affiliations:** Department of Cardiac and Vascular Surgery, University Hospital Frankfurt and Goethe University Frankfurt, Frankfurt am Main, Germany

**Keywords:** endoscopic approach, minimally invasive mitral valve repair, minimally invasive mitral valve surgery, mitral valve repair, mitral valve surgery

## Abstract

**Introduction:**

Mitral valve surgery has evolved substantially, with a clear shift towards minimally invasive approaches. This study examines clinical outcomes associated with two established techniques: standard minimally invasive mitral valve surgery (ST) and the endoscopic (EMIC) approach. While benefits such as faster recovery, improved cosmetic outcomes, reduced postoperative pain, and less blood loss have been reported for both, direct comparative data between these two techniques remain limited.

**Methods:**

We included 688 consecutive patients who underwent minimally invasive mitral valve surgery between 2018 and 2024 at our hospital. Amongst them, 514 patients (74,7%) were treated using the standard-MIC approach and 174 (25,3%) using the EMIC technique. Univariate analyses were performed to explore associations between surgical approach and relevant outcome parameters. Variables with a *p*-value < 0.05 were subsequently included in multivariate logistic regression analyses.

**Results:**

In the EMIC group, longer operative times, extended extracorporeal circulation time [169 ± 47 min (EMIC) vs. 132 ± 39 min; *p* < 0.001] and aortic cross-clamp time (88 ± 25 min vs. 73 ± 23 min; *p* < 0.001) were observed. Postoperative renal replacement therapy [10.9% (EMIC) vs. 4.3% (ST); *p* = 0.003] and pericardial effusion [11.7% (EMIC) vs. 5.3% (ST); *p* = 0.008] occurred more frequently in the EMIC group. 30-day mortality was 2.9% (EMIC) vs. 1% (ST), *p* = n.s. No statistically significant difference was observed regarding other major outcome parameters.

**Discussion:**

Minimally invasive mitral valve surgery can be safely performed using a standard minimally invasive or an endoscopic approach when performed by experienced surgeons. Despite longer operative times, the EMIC approach was feasible and demonstrated acceptable short-term safety within our institutional program, supporting its further clinical implementation.

## 1 Introduction

Minimally invasive mitral valve surgery (MIMVS) has evolved as the standard approach for mitral valve surgery (1). The shift toward less invasive access aimed to reduce surgical trauma while preserving the quality of mitral valve repair and its long-term durability. Initially performed through a right mini-thoracotomy under direct vision, MIMVS has evolved through continuous technical refinements, including the adoption of endoscopic visualization and ultrasound-guided percutaneous femoral cannulation for cardiopulmonary bypass (2). More recently, the fully endoscopic technique has emerged, utilizing three-dimensional visualization and smaller incisions, such as the so-called “nipple-cut.” Together, these developments reflect the ongoing evolution toward increasingly less invasive and patient-tailored approaches in mitral valve surgery (3, 4).

Multiple studies have demonstrated that MIMVS performed under direct vision is a safe and effective alternative to median sternotomy, offering potential advantages such as improved cosmetic outcomes, reduced postoperative pain, faster recovery, and less blood loss (5–7). The endoscopic MIMVS has likewise been reported as safe and feasible (8–10); however, direct head-to-head comparisons between the standardized right mini-thoracotomy and the endoscopic technique are lacking. Therefore, systematic analyses within single high-volume centers are needed to determine whether the endoscopic approach offers acceptable outcomes, and to identify risk factors that may influence early postoperative results.

This study aimed to compare clinical outcomes of standard minimally invasive mitral valve surgery (ST) vs. the endoscopic (EMIC) approach.

## 2 Materials and methods

### 2.1 Study population

Between January 2018 and June 2024, 2,266 patients underwent mitral valve surgery at the Department of Cardiac Surgery, University Hospital Frankfurt. Of these, 688 patients with isolated mitral valve disease were treated through a minimally invasive approach and were included in the study. Amongst them, 514 received a standard minimally invasive approach via right lateral minithoracotomy under direct vision, and 174 underwent a EMIC procedure. The decision on surgical access was made independently of the planned technique and followed institutional protocols. All cases were evaluated in the interdisciplinary heart team, taking age, comorbidities, and overall risk into account, without predefined allocation criteria for the surgical approach. Patients undergoing non-isolated mitral valve procedures, combined cardiac surgeries, or mitral valve surgery performed via alternative surgical approaches, as well as patients who refused consent for data use, were not included in the study population.

### 2.2 Surgical techniques

Both minimally invasive techniques have been previously described in the literature (11, 12). In summary, cardiopulmonary bypass was established via femoro-femoral cannulation, either percutaneously under ultrasound guidance using the Seldinger technique or by surgical exposure of the femoral vessels. Surgery was then performed through a right lateral mini-thoracotomy in the fourth intercostal space using a soft-tissue retractor. The Chitwood clamp and endoscope were introduced through small auxiliary ports in the third intercostal space (13).

**Figure 1:**
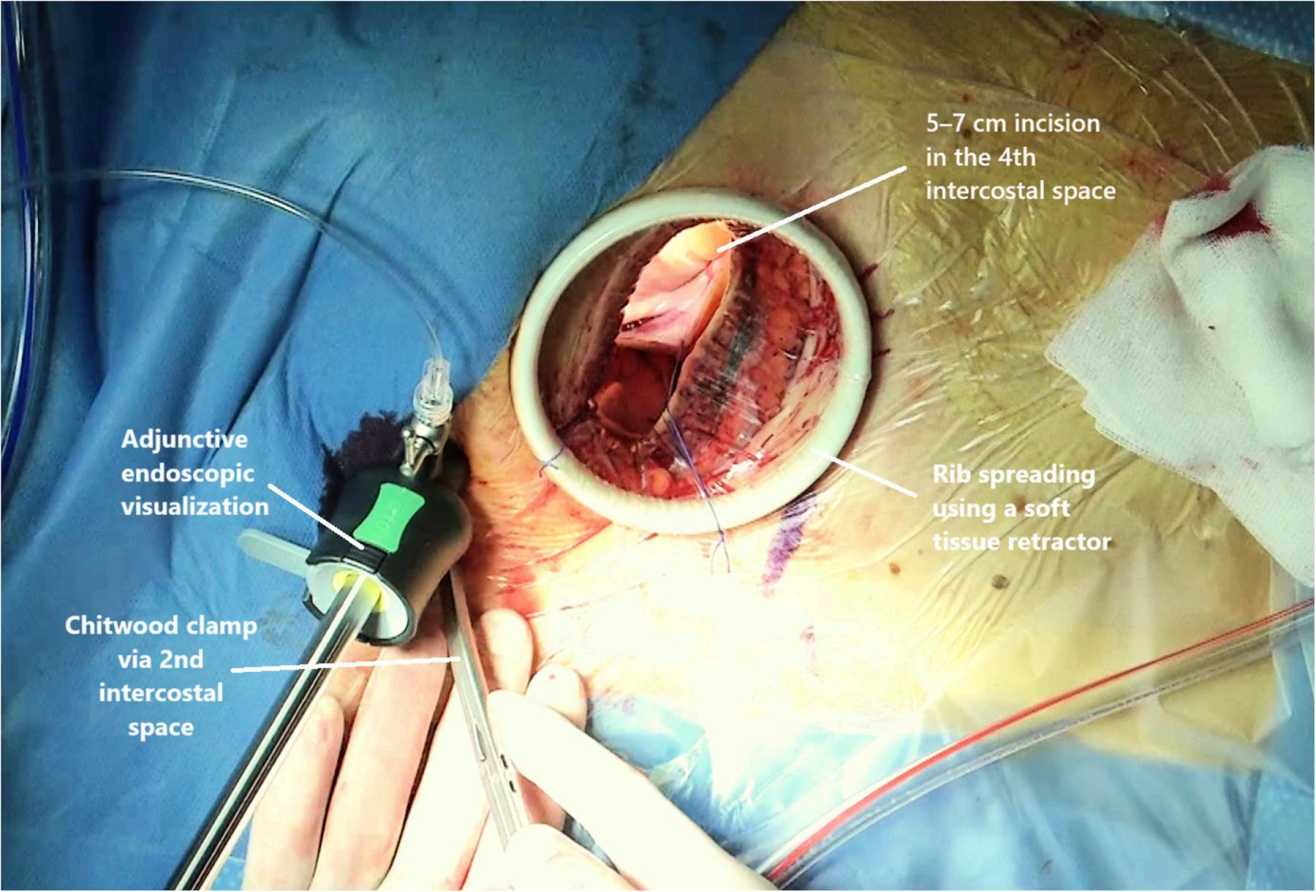
ST surgical access and setup.

In the standard-MIC cohort, the mitral valve was visualized directly under thoracotomy (Figure 1), whereas with the EMIC technique a three-dimensional camera system was used. The left atrium was opened through the interatrial groove (Waterston's groove) to provide optimal exposure of the mitral valve with both approaches.

**Figure 2:**
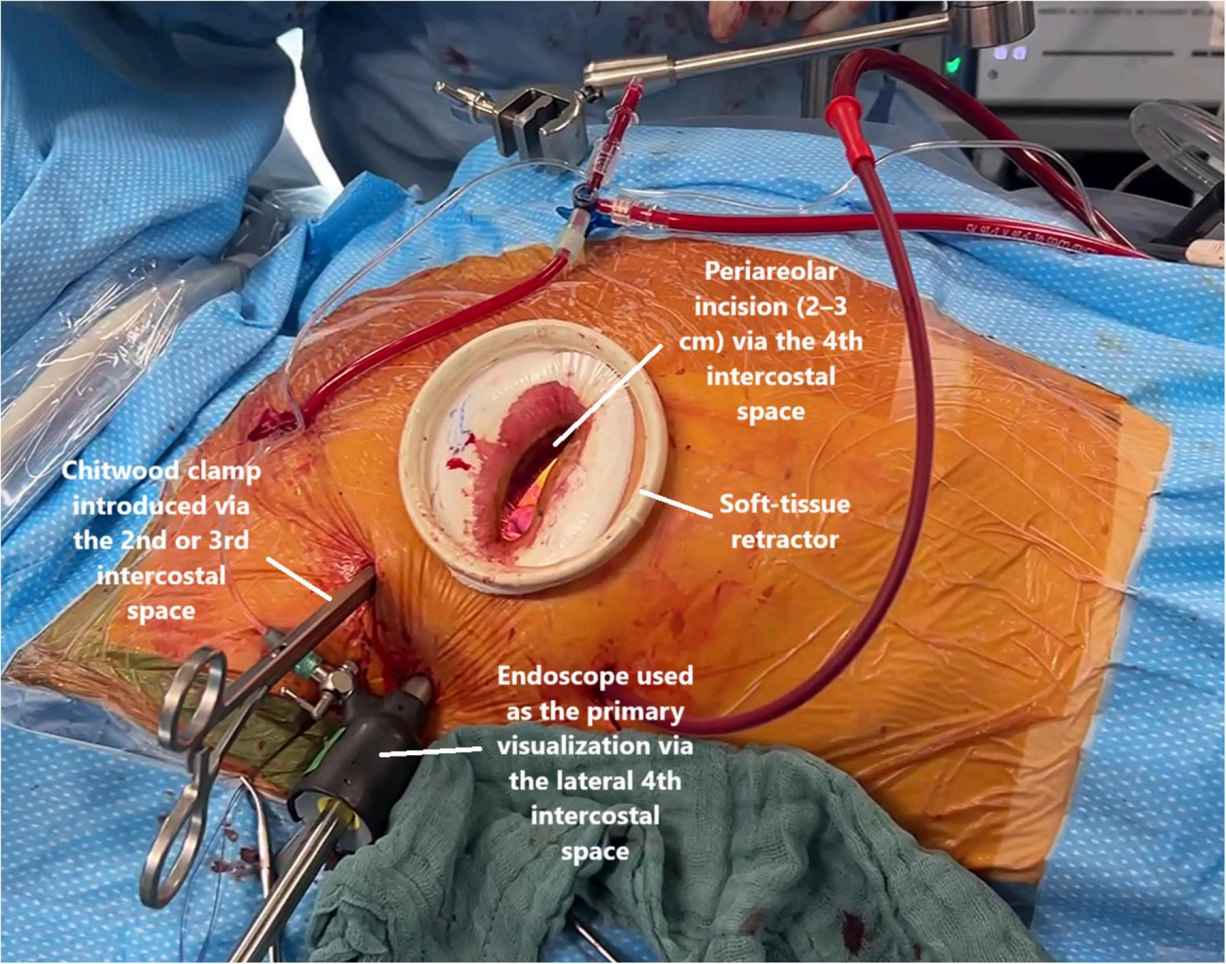
EMIC surgical access and setup.

The vast majority of procedures were performed by the same two experienced surgeons with established expertise in minimally invasive mitral valve surgery. Both surgeons were experienced with the standard minimally invasive technique. From the time of inclusion onwards, one of them performed all his operations (all comers) using an endoscopic approach with indirect visualization of the mitral valve through the camera, whereas the other performed mitral valve repair using direct vision, the camera was used in adion only. In the endoscopic cohort, access was achieved via a lateral right mini-thoracotomy (4–5 cm) or a periareolar incision (2–3 cm) through the fourth intercostal space, using a soft-tissue retractor (without rib spreading) and endoscopic visualization via a lateral fourth intercostal port with CO_2_ insufflation. The choice of a periareolar (“nipple-cut”) incision was based on patient-specific anatomical factors, including body habitus, sex, areolar diameter, and breast size (Figure 2).

Mitral valve reconstruction consisted of restoring normal leaflet motion and achieving annular stabilization. Accordingly, annuloplasty was performed in all repairs using flexible or rigid rings, with the size determined according to the anterior leaflet dimensions (14). Leaflet repair was achieved using triangular or quadrangular resection, sliding plasty, and artificial chordal replacement with expanded polytetrafluoroethylene (ePTFE, Gore-Tex) sutures using the Loop technique. When reconstruction was not feasible, valve replacement was carried out using biological prostheses (Carpentier-Edwards, Edwards Lifesciences; Epic™, Abbott) or mechanical valves (Open Pivot™, Medtronic).

### 2.3 Study design and data acquisition

This single-center cohort study was conducted at the Department of Cardiac Surgery, University Hospital Frankfurt, Germany. All analyses were performed according to an intention-to-treat principle, with patients assigned to groups based on the initially planned minimally invasive access strategy, irrespective of whether mitral valve repair or replacement was ultimately performed. Both valve repair and replacement procedures, as well as minor concomitant interventions, were analyzed. Preoperative data, including baseline characteristics and echocardiographic findings, were obtained from referring cardiologists or from the institutional outpatient clinic. Intraoperative data were extracted from surgical reports, and postoperative information was collected from electronic patient records and discharge summaries. Data were retrieved using the hospital systems Orbis and MetaVision, transferred into a dedicated Microsoft Access database, and subsequently exported to Microsoft Excel for statistical evaluation.

### 2.4 Ethics statement

This study was conducted in accordance with the Declaration of Helsinki (as revised in 2013). This study was approved by the local Ethic Committee of University Hospital Frankfurt (No. 79/13), and written informed consent was waived due to the retrospective nature of this study.

### 2.5 Statistical analysis

Normal distribution of continuous variables was assessed using the Shapiro–Wilk test. Continuous data are presented as mean ± standard deviation or median (interquartile range), as appropriate. Categorical variables are expressed as counts and percentages and were compared using the Chi-square test or Fisher's exact test when expected cell frequencies were <5. Univariate analyses were performed to identify potential predictors, and variables with a *p*-value < 0.05 were subsequently included in multivariate logistic regression analyses. Early mortality was assessed descriptively as 30-day all-cause mortality. All statistical analyses were conducted using IBM SPSS Statistics, version 29.0 (IBM Corp., Armonk, NY, USA). Figures and tables were generated with Microsoft Excel 2021 and Jamovi. A two-sided *p*-value < 0.05 was considered statistically significant. The primary endpoint of the study was a 30-day major adverse outcome, defined as the composite of all-cause mortality or stroke. All other perioperative and postoperative outcomes were considered secondary endpoints. Given the retrospective and non-randomized study design, the absence of propensity-based adjustment, and the analysis of multiple secondary endpoints, all analyses were performed in a descriptive and exploratory manner and were not intended to support inferential comparisons between surgical approach.

Clinical outcomes were defined as follows: low-cardiac-output syndrome (LCOS) was defined as postoperative hemodynamic instability requiring inotropic/vasopressor therapy or mechanical circulatory support (ECLS or ECMO). Postoperative myocardial infarction was defined based on standard clinical criteria documented in the medical records. Neurological events were defined as any new postoperative neurological complication and categorized as transient ischemic attack, minor or major stroke, coma, or other neurological complications (e.g., delirium).

## 3 Results

### 3.1 Patients characteristics

Baseline characteristics are provided in Table 1. Patients had a mean age of 61.9 ± 11.9 years and were predominantly male, with 36% being female. Patients in the EMIC group more frequently presented with advanced heart failure (NYHA ≥ III, *p* = 0.023). Non-sinus rhythm was defined as the presence of paroxysmal or chronic atrial fibrillation, including cases with atrial fibrillation despite pacemaker rhythm, with no significant difference between groups. Previous relevant cardiac surgery was defined as any prior mitral valve reconstruction or valvuloplasty procedure. Apart from NYHA class and previous cardiac surgery, baseline characteristics were comparable between groups.

**Table 1:** Demographics.

Parameters	All patients (*n* = 688)	EMIC (*n* = 174)	ST (*n* = 514)	*P*-value
Age (years)	61.9 ± 11.9 [63 (16)]	60.5 ± 12 [61.5 (16)]	62.3 ± 12 [63 (17)]	0.132
Female (*n*.%)	248 (36%)	65 (26.2%)	183 (73.8%)	0.715
BMI	25.7 ± 4.8 [25 (12)]	25.9 ± 5 [25 (12)]	25.6 ± 5 [25 (14)]	0.314
NYHA ≥ III(*n*.%)	258 (37.7%)	78 (45.1%)	180 (35.2%)	0.023
sPAP (mmHg)	38.8 ± 15 [35 (20)]	40 ± 16 [37 (23)]	38 ± 14 [35 (19)]	0.257
Non-sinus rhythm (*n*. %)	243 (35.3%)	53 (30.5%)	190 (37.0%)	0.142
Previous relevant cardiac surgery	37 (5.4%)	16 (9.2%)	21 (4.1%)	0.018
COPD Moderate–Severe (2–4)	38 (5.6%)	6 (3.5%)	32 (6.3%)	0.247
LV function	62.7 ± 9 [63 (11)]	63 ± 9 [63 (12)]	62.6 ± 10 [63 (10)]	0.811
History of stroke (*n*,%)	37 (5.4%)	11 (6.4%)	26 (5.1%)	0.558
EuroSCORE II	2.3 ± 2.3 [1.35 (2)]	2.4 ± 2.22 [1.4 (2)]	2.305 ± 2.3 [1.3(1,8)]	0.565

Values are *n* (%) or mean ± standard deviation [median (interquartile range)].

EMIC, endoscopic MIMVS; ST, standard MIMVS; BMI, body mass index (kg/m^2^); NYHA, New York Heart Association functional classification; sPAP, systolic pulmonary artery pressure; COPD, chronic obstructive pulmonary disease, classified according to the Global Initiative for Chronic Obstructive Lung Disease (GOLD) scale; LV function, left ventricular ejection fraction; EuroSCORE II, European System for Cardiac Operative Risk Evaluation II.

### 3.2 Intraoperative data

Intraoperative data are summarized in Table 2. Cardiopulmonary bypass and aortic cross-clamp times were significantly longer in the endoscopic group (169 ± 47 min and 88 ± 25 min, respectively) compared with the ST group (132 ± 39 min and 73 ± 23 min; both *p* < 0.001). Among 688 patients treated with MIMVS, mitral valve repair was performed in 480 cases, corresponding to an overall repair rate of 69.8%, while 208 patients (30.2%) underwent valve replacement. Percutaneous cannulation was used in 96.7% of all cases, with no significant difference between groups.

**Table 2:** Intraoperative results.

Parameters	All patients (*n* = 688)	EMIC (*n* = 174)	ST (*n* = 514)	*P*-value
Operative times (min)				
Cardio-pulmonary bypass time	141.23 ± 44 [131 (51)]	169 ± 47 [160 (60)]	132 ± 39 [122 (39)]	<0.001
Aortic cross-clamp time	76.63 ± 24 [72 (29)]	88 ± 25 [86 (29)]	73 ± 23 [68 (27)]	<0.001
Mitral valve Repair (*n*,%)	480 (69.8%)	123 (70.7%)	357 (69.5%)	0.776
Mitral valve Replacement (*n*,%)	208 (30.2%)	51 (29.3%)	157 (30.5%)	0.035
Conversion to sternotomy (*n*,%)	15 (2.2%)	5 (2.9%)	10 (1.9%)	0.547
Percutaneous cannulation (*n*,%)	665 (96.7%)	170 (97.7%)	495 (96.3%)	0.471
Primary MR (*n*,%)	606 (88.1%)	159 (91.4%)	447 (87.0%)	< 0.001
Degenerative (*n*.%)	412 (59.9%)	88 (50.6%)	324 (63.0%)	
Barlow's disease (*n*.%)	81 (11.8%)	33 (19.0%)	48 (9.3%)	
Other (*n*.%)	95 (13,8%)	23 (13.2)	70 (13.6%)	

Values are *n* (%) or mean ± standard deviation [median (interquartile range)].

EMIC, endoscopic MIMVS; ST, standard MIMVS. Other etiologies include acute and chronic endocarditis, rheumatic, and calcific mitral valve disease. Detailed subclassification is provided in the Supplementary Material.

### 3.3 Postoperative outcomes

Postoperative outcomes are summarized in Table 3. A total of 59 patients (8.6%) required re-thoracotomy. Most re-explorations were performed via re-mini thoracotomy for postoperative bleeding or hematoma evacuation. Other indications included pericardial effusion and, in isolated cases, pericardial tamponade. Four patients required multiple re-operations. There was no significant difference between the two groups (*p* = 0.449). Gastrointestinal complications occurred in 9 patients (1.3%), mainly due to postoperative bleeding, while two cases were caused by intestinal ischemia. Neurological complications were observed in 6 patients (0.9%), including two transient ischemic attacks, two strokes, one case of stroke-related postoperative coma, and one case of postoperative coma unrelated to cerebrovascular events. Pericardial effusion was significantly more frequent in the EMIC group (11.7%; *p* = 0.008), and the need for renal replacement therapy was also higher in this group (10.9%; *p* = 0.003). Residual mitral regurgitation was defined as grade ≥ 2 and did not differ significantly between groups. Low-cardiac-output syndrome occurred in 13 patients (1.9%), of whom two required ECLS therapy and eight could be treated conservatively. Ventilation time differed significantly between groups (29 ± 69 h vs. 22 ± 52 h; *p* < 0.001). Causes of early mortality included low-cardiac-output syndrome in two patients, sepsis and multi-organ failure in three patients, and one case of severe neurological injury. Thirty-day mortality was 2.9% in the EMIC group and 1.0% in the ST group and did not differ significantly between groups (*p* = 0.133).

**Table 3:** Postoperative outcomes.

Parameters	All patients (*n* = 688)	EMIC (*n* = 174)	ST (*n* = 514)	*P*-value
Re-thoracotomy	59 (8.6%)	11 (6.3%)	45 (9.3%)	0.273
Myocardial infarction (*n*,%)	4 (0.6%)	2 (1.1%)	2 (0.4%)	0.266
Reintubation (*n*,%)	42 (6.1%)	9 (5.2%)	33 (6.4%)	0,714
Neurological complications	6 (0.9%)	0 (0.0%)	6 (1.2%)	0.346
Gastrointestinal complications	9 (1.3%)	1 (0.6)	8 (1.6%)	0.220
Pericardial effusion (*n*,%)	47 (6.9%)	20 (11.7%)	27 (5.3%)	0.008
Renal replacement therapy (*n*,%)	41 (6.0%)	19 (10.9%)	22 (4.3%)	0.003
Pacemaker implantation (*n*,%)	27 (3.9%)	5 (2.9%)	22 (4.3%)	0.503
Atrial fibrillation (*n*,%)	162 (23.5%)	38 (21.8%)	124 (24.1%)	0.606
Residual regurgitation ≥ 2 (*n*,%)	23 (3.4%)	8 (4,7%)	15 (2.9%)	0.327
LCOS treated by ECMO	13 (1.9%)	5 (2.9%)	8 (1.6%)	0.467
Ventilation time (hours)	23,7 ± 57 [10 (6)]	29 ± 69 [(12)]	22 ± 52 [9 (4)]	<0.001
ICU stay (days)	2.4 ± 4.4 [1 (1)]	2.8 ± 5 [1 (2)]	2.2 ± 4 [1 (1)]	0.076
Hospital stay (days)	10,3 ± 8.2 [8 (4)]	10.5 ± 10 [8 (4)]	10.2 ± 8 [8 (4)]	0.791
Early mortality (30 days) (*n*,%)	10 (1.5%)	5 (2.9%)	5 (1.0%)	0.133

Values are *n* (%) or mean ± standard deviation [median (interquartile range)].

EMIC, total-endoscopic MIMVS; ST, standard MIMVS, ECMO, extracorporeal membrane oxygenation; LCOS, low-cardiac-output syndrome; ICU, intensive care unit; MIMVS, minimally invasive mitral valve surgery.

## 4 Discussion

This study represents a detailed analysis of two established minimally invasive mitral valve surgery (MIMVS) techniques at, a high-volume institution. Previous studies have compared the ST appraoch with median sternotomy, demonstrating satisfactory short- and long-term outcomes (15–19). Likewise, several reports have confirmed the safety and feasibility of the EMIC approach (20–24). However, direct comparisons between these two minimally invasive approaches remain limited. Despite longer cardiopulmonary bypass and aortic cross-clamp times in the EMIC group, perioperative morbidity and early mortality remained within the expected range of reported outcomes.

Operative metrics, including cardiopulmonary bypass (CPB) and aortic cross-clamp (ACC) times, provide key indicators of technical efficiency and procedural complexity. Previous studies have reported comparable operative durations between ST and EMIC (25, 26). Comparable results were reported by Chen et al. (mean ACC ≈ 102 min) and Squiccimarro et al. (median CPB ≈ 126 min, ACC ≈ 93 min). In the present study, CPB and ACC times were longer in the EMIC group, which likely reflects the increased technical demands of the endoscopic setup and the early implementation phase of the program rather than a procedural limitation. Learning-curve effects during the adoption of minimally invasive techniques have been well described (27, 28). The EMIC approach was introduced at our center in 2019, and this study includes the first patients operated using this approach. The longer operative time should therefore be interpreted in a descriptive context as a reflection of expected learning-curve effects and procedural refinement rather than as a technical limitation. Accordingly, cardiopulmonary bypass and aortic cross-clamp times decreased over the study period in the endoscopic cohort (CPB: 216 to 159 min; ACC: 110.6 to 84.9 min), with a similar but less pronounced trend observed in the standard minimally invasive cohort.

Longer operative and anesthesia durations in the EMIC group were accompanied by slightly prolonged ventilation times. Gammie et al. observed a median ventilation time of approximately 6 h following standard MIMVS in more than 4,000 patients (29), whereas Squiccimarro et al. reported a median of 5 h after EMIC repair (25). Such differences are likely influenced by perioperative management strategies, extubation protocols, and institutional standardization rather than by the surgical approach itself. A modest prolongation of ventilation time may also be expected during the early adoption phase of a new technique (30).

Despite longer operative times in the EMIC group, perioperative safety and postoperative recovery were observed to be similar across approaches. Conversion to sternotomy was rare, with no significant difference between groups (2.9% vs. 1.9%) and rates consistent with previous reports (17). Reported causes included bleeding, limited exposure, or right ventricular injury, all of which are well documented during the early learning phase of minimally invasive programs (28). The purely endoscopic visualization and the absence of direct tactile feedback increase the technical demand of the procedure, particularly in complex pathologies or challenging anatomy. Nevertheless, conversions remained infrequent in both groups, confirming the overall procedural safety of both approaches.

Pericardial effusion occurred in 11.7% (EMIC) vs. 5.3% (ST). This represents a clinically relevant difference and may be considered a potential disadvantage of the endoscopic approach, particularly during early adoption phases. This difference may in part be attributed to the more frequent use of intraoperative pericardial drainage in the ST group, which was performed only in selected cases in the endoscopic cohort. The incidence of renal replacement therapy was higher in the EMIC-group. This may be influenced by patient-specific risk factors, including preexisting renal impairment, as well as longer cardiopulmonary bypass duration. Rates of re-thoracotomy for bleeding were similar between groups and consistent with previously published data. Interestingly, a gradual decline in reoperation rates was observed over the study period, likely reflecting increasing procedural standardization and improved intraoperative hemostasis. Neurological complications were rare and showed no significant difference between approaches. All reported complication rates remained within the expected range from the literature (25, 31, 32), supporting that both minimally invasive techniques are safe and reproducible in experienced centers.

In line with the overall low complication rates, postoperative recovery was favorable in both groups, reflecting the minimally invasive nature of the procedures. Wound healing complications were rare, occurring in less than 2% of all patients (thoracic 0.2%, groin 1.4%), and were comparable to rates reported by Chen et al. (33) and Cohn et al. (34). Patients in both groups benefited from fast recovery patterns typically associated with MIMVS (35). Intensive care unit stay was short, with a median of one day in both groups, consistent with previous studies (32, 33). The median hospital stay was eight days for both approaches, in line with the German standards and the relevant literature (22, 36). A progressive decline in hospital stay duration over the study period (from 14 to 9 days) further reflects increasing standardization, institutional efficiency, and gained procedural experience. Together, these findings underline the reproducibility and efficiency of both minimally invasive techniques in facilitating stable postoperative recovery.

Another important parameter for assessing operative success is the evaluation of residual mitral regurgitation. In this study, residual regurgitation was defined as ≥ grade II, resulting in rates of 4.7% in the EMIC group and 2.9% in the ST group. These findings are in line with previously published data from large minimally invasive series. Glauber et al. reported moderate residual MR in only 0.08% of patients at discharge after surgical revision, corresponding to an initial early postoperative failure rate of 2.5% before reoperation (37). Similarly, Seeburger et al. observed a 1.6% rate of early repair failure requiring conversion or reoperation prior to discharge (38). Together, these results highlight that early residual MR after minimally invasive mitral valve repair remains infrequent in high-volume centres.

In this study, early mortality, defined as 30-day mortality, was 2.9% (EMIC) vs. 1.0% (ST), this did not reach statistical significance (*p* = 0.133). These rates are within the range reported in the literature, with Squiccimarro et al. (25) documenting 1%, Chen et al. 0.5% (33), and Grossi et al. 4.2% (31). Galloway et al. and Modi et al. reported early mortalities of 2.2% and 2.1–4.6%, respectively (22, 39). In the present series, all deaths in the TE group occurred after valve replacement rather than repair, reflecting the higher complexity of these patients. In four cases, intraoperative conversion from an intended repair to valve replacement was necessary due to leaflet or annular pathology. Early mortality occurred infrequently in both groups and remained within the range reported in previous minimally invasive mitral valve surgery series. In the present cohort, deaths were predominantly observed in patients who experienced major perioperative complications, including myocardial infarction, gastrointestinal or neurological events, and low-cardiac-output syndrome. Operative experience, patient selection, and intraoperative decision-making remain key determinants of early outcome.

### 4.1 Limitations

This study has several limitations that should be considered when interpreting the results. The retrospective design carries an inherent risk of bias, particularly due to the absence of randomization. A relevant temporal bias must be acknowledged, as the endoscopic approach was introduced later and includes the early adoption phase, whereas the standard approach represents a longer-established technique. Baseline differences between both groups, including a higher NYHA functional class and a prevalence of redo surgery in the EMIC group, further limit direct comparability between both approaches. A systematic long-term follow-up beyond the 30-day mortality period could not be performed within the given study framework and should be addressed in future investigations. Potential examiner bias in preoperative echocardiographic assessment must also be taken into account, as examinations were performed under varying conditions and influenced by individual operator experiences. In some cases, discrepancies between preoperative findings and intraoperative assessment of valve etiology were observed. Another limitation is the unequal distribution of patients between groups, which reduces comparability and increases the potential for distortion. Due to the very low number of early mortality events, time-to-event analyses were not performed. The absence of formal learning curve adjustment represents an additional limitation of the present study. Future studies could apply statistical methods such as propensity score matching or inverse probability weighting to improve the balance for even better comparison. Finally, differences in surgical experience between operators may lead to an additional source of variability.

### 4.2 Conclusion

The present study demonstrates the feasibility of the EMIC approach within a high-volume centre performing routine minimally invasive mitral valve surgery. The observed perioperative and early postoperative outcomes provide descriptive insight into its application during institutional implementation. Future prospective studies are warranted to evaluate long-term outcomes and patient-centred parameters such as recovery and quality of life.

## Data Availability

The original contributions presented in the study are included in the article/Supplementary Material, further inquiries can be directed to the corresponding author/s.
