# Supplementary material for: Minimally invasive mitral valve surgery: standard vs. endoscopic approach
Source: Front Cardiovasc Med. 2026 Feb 19;13:1736030. doi: 10.3389/fcvm.2026.1736030 (PMC12960477; doi:10.3389/fcvm.2026.1736030)
Supplement: Supplementary file 1 [file Datasheet1.docx]

Supplementary Material

# Supplementary Data

# Supplementary Figures and Tables

Table S1: Multivariate Cox regression analysis for non-significant predictors of 30-day mortality

| Parameters | P-value | Hazard Ratio (HR) | 95% Confidence Interval  (Lower limit – Upper limit) | |
| --- | --- | --- | --- | --- |
| Left ventricular function | 0.349 | 2.385 | 0.387 | 14.696 |
| Peripheral arterial disease (PAD) | 0.290 | 7.752 | 0.174 | 345.129 |
| Cardiopulmonary bypass time (min) | 0.612 | 1.011 | 0.969 | 1.055 |
| Aortic cross-clamp time (min) | 0.899 | 0.995 | 0.920 | 1.076 |
| Additional valve procedure | 0.654 | 0.690 | 0.136 | 3.502 |
| Repair vs. replacement | 0.571 | 0.033 | 0.000 | 4245.556 |
| Additional procedure (ASD closure) | 0.664 | 2.016 | 0.086 | 47.418 |
| Annuloplasty ring reconstruction | 0.360 | 0.157 | 0.003 | 8.300 |
| Left atrial appendage closure | 0.221 | 3.610 | 0.462 | 28.244 |
| Intraoperative complications | 0.157 | 9.089 | 0.427 | 193.370 |
| Re-thoracotomy | 0.402 | 0.338 | 0.027 | 4.264 |
| Re-intubation | 0.724 | 1.676 | 0.098 | 29.325 |
| Renal replacement therapy | 0.476 | 0.293 | 0.010 | 8.540 |
| Ventilation time (hours) | 0.093 | 0987 | 0.972 | 1.002 |

**Table S2: Etiology and pathology of primary mitral regurgitation**

| Parameters | All patients  (n=688) | Total-endoscopic  (n=174) | Standard MIMVS  (n=514) | P-value |
| --- | --- | --- | --- | --- |
| Primary (n,%) | 606 (88.1%) | 159 (91.4%) | 447(87.0%) | < 0.001 |
| Etiology (primary MR) |  |  |  |  |
| Degenerative (n,%) | 412 (59.9%) | 88 (50.6%) | 324 (63.0%) |  |
| Barlow’s disease (n,%) | 81 (11.8%) | 33 (19.0%) | 48 (9.3%) |  |
| Acute endocarditis (n,%) | 26 (3.8%) | 4 (2.3%) | 22 (4.3%) |  |
| Chronic endocarditis(n,%) | 12 (1.7%) | 3 (1.7%) | 9 (1.8%) |  |
| Rheumatic (n,%) | 35 (5.1%) | 11 (6.3%) | 24 (5.4%) |  |
| Calcific (n,%) | 22 (3.2%) | 5(2.9%) | 15 (2.9%) |  |
| Other: |  |  |  |  |
| Congenital (n,%) | 2 (0.3%) | 7 (4.0%) | 0 (0.0%) |  |
| Previous mitraClip (n,%) | 6 (0.9%) | 2 (1.1%) | 4 (0.8%) |  |
| Previous mitral valve repair (n,%) | 10 (1.7%) | 9 (5.2%) | 1 (0.2%) |  |
| Pathology of primary MR (n,%) |  |  |  | <0.001 |
| Anterior leaflet prolapse (n,%) | 40 (5.8%) | 11 (6,3%) | 29 (5.6%) |  |
| Posterior leaflet prolapse (n,%) | 390 (56.7%) | 88 (50.6%) | 302 (58.8%) |  |
| Bileaflet prolapse (n,%) | 79 (11.5%) | 32 (18.4%) | 47(9.1%) |  |
| Indentation (n,%) | 7 (1.0%) | 0(0.0%) | 7 (1.4%) |  |
| Leaflet perforation (n,%) | 15 (2.2%) | 6 (3.4%) | 9 (1.8%) |  |
| Values are n (%) | | | | |

**Table S2: Etiology and pathology of primary secondary regurgitation**

| Parameters | All patients  (n=688) | Total-endoscopic  (n=174) | Standard MIMVS  (n=514) | P-value |
| --- | --- | --- | --- | --- |
| **Secondary** (n,%) | 64 (9.3%) | 12 (6.9%) | 52 (10.1%) | <0.001 |
| **Etiology (secondary MR)** |  |  |  |  |
| Atrial (n,%) | 24 (3.5%) | 3 (1.7%) | 21 (4.1%) |  |
| Secondary cardiomyopathy (CMP) (n,%) | 29 (4.2%) | 5 (2.9%) | 24 (4.7%) |  |
| Secondary coronary artery disease (CAD) (n,%) | 9 (1.3%) | 2 (1.1%) | 7 (1.4%) |  |
| **Other:** |  |  |  |  |
| Previous annuloplasty ring dehiscence (n,%) | 2 (0.3%) | 2 (1.1%) | 0 (0.0%) |  |
| **Pathology of secondary MR** |  |  |  | <0.001 |
| Isolated annular dilatation (n,%) | 62 (9.0%) | 10 (5.7%) | 52 (10.1%) |  |
| Previous annuloplasty ring dehiscence (n,%) | 2 (0,3%) | 2 (1.1%) | (0.0%) |  |
| Values are n (%) | | | | |

Table S3: Detailed intraoperative Techiques

| Parameters | All patients  (n=688) | Total-endoscopic  (n=174) | Standard MIMVS  (n=514) | P-value |
| --- | --- | --- | --- | --- |
| **Additional procedures** |  |  |  |  |
| Coronary artery bypass grafting (n,%) | 6(0.9%) | 2(1.1%) | 4(0.8%) | 0.646 |
| Ablation procedure (n,%) |  |  |  | <0.001 |
| None (n,%) | 471(68.5%) | 124(71.3%) | 347(67.5%) |  |
| Endocardial cryoablation (n,%) | 154(22.4%) | 4(2.3%) | 150(29.2%) |  |
| Endo- and epicardial cryoablation (n,%) | 63(9.2%) | 46(26.4%) | 17(3.3%) |  |
| Additional valve procedures (n,%) |  |  |  | 0.106 |
| None (n,%) | 599(87.1%) | 146(83.9%) | 453(88.1%) |  |
| Aortic valve replacement (n,%) | 1(0.1%) | 1(0.6%) | 0(0.0%) |  |
| Aortic valve repair (n,%) | 1(0.1%) | 1(0.6%) | 0(0.0%) |  |
| Aortic procedure (n,%) | 1(0.1%) | 0(0.0%) | 1(0.2%) |  |
| Tricuspid valve repair (n,%) | 86(12.5%) | 26(14.9%) | 60(11.7%) |  |
| Atrial septal defect closure (n,%) | 3(0.4%) | 1(0.6%) | 2(0.4%) | <0.001 |
| Annuloplasty (n,%) |  |  |  | <0.001 |
| None (n,%) | 195(28.3%) | 44(25.3%) | 151(29.4%) |  |
| Physio ring (n,%) | 381(55.4%) | 52(29.9%) | 329(64.0%) |  |
| ETlogix ring (n,%) | 6(0.9%) | 0(0.0%) | 6(1.2%) |  |
| Memo 4D annuloplasty ring (n,%) | 102(14.8%) | 78(44.8%) | 24(4.7%) |  |
| Cosgrove band (n,%) | 4(0.6%) | 0(0.0%) | 4(0.8%) |  |
| Values are n (%) | | | | |

**
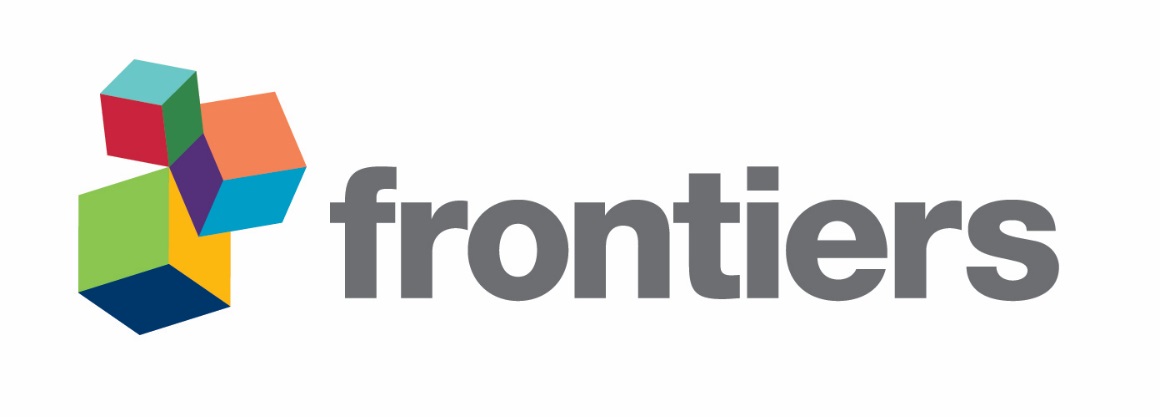
**
